# Barriers to Workforce-Driven Innovation in Healthcare

**DOI:** 10.7759/cureus.72316

**Published:** 2024-10-24

**Authors:** Alaa I Al-Saleem, Manal K Aldakheel

**Affiliations:** 1 Nursing, King Faisal Specialist Hospital and Research Centre, Riyadh, SAU; 2 Nursing, Dr. Sulaiman Al Habib Medical Group, Riyadh, SAU

**Keywords:** barriers, healthcare, innovation, solutions, workforce

## Abstract

Healthcare systems are continually developing new ways of delivering care in pursuit of quality improvement, increasing patient and provider satisfaction, and enhancing efficiency. This review measures the barriers to workforce-driven innovation in healthcare, a crucial yet underexamined area of study. Research into workforce-driven innovation in healthcare identifies several key barriers and solutions. Major impediments include a lack of knowledge and resources, as well as financial constraints, which hinder the implementation of innovations. Research findings from different regions enumerate organizational capability, leadership quality, and the adequacy of human resource management as influencing factors for innovation. Poor leadership, lack of inter- and cross-organizational learning, and structural barriers related to inadequate communication and formal forums also hinder progress. In addition, the resistance to change and the inability to engage employees effectively further obstruct innovation efforts. These issues can be addressed through the creation of a supportive innovation environment, enhancing training and development, and improving communication networks. Despite thorough database searches, the emerging nature of this topic has resulted in limited literature, restricting a comprehensive comparison of studies. Future research studies should be longitudinal in design, from diverse geographic contexts, and focus on the effects of emerging technologies to comprehensively understand these barriers and develop effective strategies for overcoming them.

## Introduction and background

In the healthcare industry, innovations drive improvements in service quality and overall well-being while continually challenging current providers and systems. Advancements in healthcare management often involve innovative organizational methods, such as improved care pathways, workforce-driven strategies, and patient-centered care models. The significant enhancement in quality of life and longevity over the past century can be credited to breakthroughs in healthcare and related areas, including hygiene and nutrition. Innovation continually enhances preventive measures [1]. Innovation can be defined as the combination of invention, adoption, and diffusion. In the healthcare sector, it involves a new idea, product, service, or care pathway that offers distinct advantages over existing practices. Effective innovations typically have two main characteristics: they are both practical and appealing [2].

Innovations involve various minor improvements organizations make to their current production methodology, products, or services. The growth in innovation has been associated with the creativity and idea generation of employees. Consequently, the rise in practice-driven processes has the potential to fundamentally transform health management systems over time [3]. The governing body within an organization must support and recognize the advancement of innovations at the micro-level. The efforts and behaviors of employees that drive incremental innovation are termed workforce-driven innovation (WDI). Explaining WDI in its basic form involves the creation and implementation of new products, processes, and ideas, including changes to organizational practices and routine job functions [4,5]. In healthcare, employees are considered the most valuable assets. Leveraging the knowledge of the workforce empowers workers and enhances the development of WDI. Effective human resource (HR) practices play a crucial role in fostering this innovation [5,6].

At the outset of technological innovations, organizations recognized that the collective innovative efforts of individual employees significantly enhanced overall organizational performance [7]. Research findings have increasingly underlined the value of bottom-up approaches in driving innovation, wherein individual employees are the source of new ideas. Accordingly, scholars have noted that such bottom-up processes allow employees from all levels of the organization to meaningfully contribute through innovative ideas that are then disseminated and adopted within the organization. Grassroots involvement in this way is very important in driving general organizational performance toward achieving successful innovation outcomes. By tapping more creativity and insights from the workforce, an organization is able to leverage a greater range of ideas and solutions, therefore maximizing efficiency and effectiveness [3,4,8].

By feeling the impact of influencing their workplace, the workers are likely to perform better and raise the overall organizational performance. Contrarily, with an increased lack of employee empowerment, an organization faces more significant risks of losing an ambitious workforce to other firms [9,10]. Most healthcare organizations, especially public ones, get their governance from top-down management that influences the innovation perspective to focus on radical instead of employee-participatory and incremental innovations. At the core of WDI is the critical tenet that every workforce, regardless of their education and position, can contribute something to the organization's innovations [3,5]. Moreover, the workforce involved in the innovations within the organizations is not employed for the reason of carrying out activities related to innovation. Instead, they are just ordinary workers who hold different positions and are operating in diverse departments [4]. There has also been a linkage of workforce participation in work-learning opportunities, such as formal workplace discussions and training, to WDI. With the activities pertaining to WDI, employees gain interpersonal awareness and practical skills, and they learn more about the organization, which may motivate them to develop changes and improvisations to work practices and processes [7]. Workplace learning through daily routines and customer interactions can promote communication and explorations among peers [3].

This review aims to explore the barriers that may hinder the effective implementation of employee-driven innovations within healthcare settings and pinpoint the importance of WDI to organizational success. WDI in healthcare is critical, given that it exploits staff creativity and insight in driving improvement related to patient care and operating efficiency. However, despite its importance, in practice, several challenges commonly face healthcare organizations in WDI, such as resistance to change, inadequate support systems, and limited resources. This review considers those barriers and provides requisite insights and recommendations for healthcare leaders and policymakers to create a supportive environment that improves workforce-driven innovation and contributes to the success and advancement of healthcare organizations.

## Review

Methodology

This study is a narrative review aimed at synthesizing the current literature on WDI in healthcare. A comprehensive literature search was conducted across multiple databases, including PubMed, Science Direct, and Cochrane, as well as Google Scholar for supplementary manual searches, on September 3, 2024. The search strategy employed a combination of relevant keywords and topic headings such as "innovation", "novel", "workforce", "employee-driven", "challenges", "barriers", and "implications". Boolean operators (e.g., AND, OR) were used to refine and combine search terms (e.g., "innovation AND healthcare AND workforce”). No restrictions were placed on publication dates or participant demographics, allowing for a broad exploration of the literature. Articles were limited to those published in English and involving human subjects.

To ensure relevance, studies were selected based on predefined inclusion and exclusion criteria. Inclusion criteria included peer-reviewed articles and original research studies that focused on workforce-driven innovation in healthcare, including barriers, facilitators, leadership interventions, or workforce empowerment. Studies involving healthcare workers, organizations, or patients were considered, particularly those addressing WDI outcomes such as changes in organizational practices, decision-making processes, or patient care improvements. Exclusion criteria included case reports, editorials, commentaries, or non-peer-reviewed literature, as well as studies not directly related to WDI or healthcare innovation.

The study selection process involved both authors independently screening the titles and abstracts of identified studies to ensure they met the inclusion criteria. Full-text articles of selected studies were reviewed in detail to assess their relevance and methodological rigor. In cases where there were disagreements between the two authors regarding the inclusion of a study, discussions were held to reach a consensus. If disagreements persisted, they were resolved collaboratively through deliberation based on the study's alignment with the review objectives.

Although this review is not systematic and does not employ formal risk of bias tools, articles were assessed for their relevance, methodology, and quality based on the study objectives. Studies with limited methodological rigor or without detailed results related to workforce-driven innovation were excluded during the selection process. This approach ensured that the review captured the most relevant and high-quality research available, providing a solid foundation for the analysis of WDI in healthcare.

Discussion

There are several challenges in creating an innovative culture in organizations to fully leverage employee-driven ideas. For example, when rapid discovery competitions are not supported by the organization, WDI may result in a series of project ideas that are short-lived because of a lack of long-term solutions. Second, locally developed innovations may provide temporary fixes only and may also fail to integrate into organizational processes. Poor documentation of such innovations may obscure the real value of WDI. The employee ideas that come from spontaneous and uncoordinated efforts are even less predictable and may break into established systems. In the healthcare sector, both implementation and persistence of innovation are particularly problematic, as this is a sector that resists change. Thus, in the case of complex organizations within healthcare institutions trying to implement a new innovation dynamic, one serious barrier is how those organizational and institutional changes are managed [11]. Knowing that setbacks can occur allows continuous learning and critical analysis to occur in organizations. Thorough debriefing on successes and failures allows for the ongoing identification of success factors and barriers, and the development of new strategies, and approaches. Organizations can achieve sustained support; however, from a wide range of stakeholders by establishing and tracking metrics of impacts on patients, employers, and health systems that show the real value of investments and new initiatives. Indeed, over time, truly effective tool and technique development to drive successful innovation can yield long-term positive changes [12]. In the light of existing literature, we discuss below some WDI barriers and propose solutions for these challenges ensuring that the implications of novel, feasible, and optimal innovations in healthcare settings are guaranteed.

Barriers

Knutsson and Lurie identified that the main obstacles to innovation were associated with the types of innovations being pursued. They identified significant challenges, such as insufficient knowledge and resources. Moreover, they noted that many regions in Sweden faced a shortage of financial resources [13]. Similarly, Renkema et al. described certain factors that constrain employee-driven innovation, including lack of resources, recognition, priority, and organizational knowledge. Also, despite the constant management expectation that the workforce would formulate ideas, most employees saw minimal possibilities for innovation. Additionally, the authors discovered that a bottom-up innovation process fully developed, initiated, and implemented by the frontline workforce was an exception. Most of the bottom-up development initiatives involved critical manager roles [8]. Further, Kelly and Young highlighted that the major challenges to innovations in healthcare involve limited budgets and rising clinical demands, as it often leads to innovative methods being the first to be sacrificed. However, embracing failure as a component of the innovation process is crucial, as there is no penalty for adhering to established practices. Yet, attempting innovative changes that fail can have significant repercussions for clinicians or the board of trusts and clinical commissioning groups [2]. Samuelson et al. also indicated that it was challenging for new employees to understand the employee-driven innovation model and see the long-term benefits. An additional challenge was staying engaged with the innovative work when it was stressful and task-oriented. Employees also reported that they were unable to pursue certain ideas due to a lack of external assistance. New employees needed help understanding the method of working with WDI. Being part of the development of work practices was a new experience for them since they had previously received top-down orders on what and how work should be done. Although they enjoyed the novel style of working, they were not accustomed to prioritizing innovative and creative work over clinical work [14].

Furthermore, employee resistance to innovation is frequently cited as one of the primary impediments to innovative change and a negative aspect of work relationships in healthcare organizations. Identifying the sources of resistance is a pressing issue for all organizations, as the rate of change always affects their competitiveness. The proposed methodology is based on quantitative modeling methods. The use of a modeling approach can assist administrators of healthcare organizations in eliminating sources of staff resistance to change, hence accelerating innovation development [15]. However, a study by Mansour and Nogues depicted that nurses were proactive and optimistic about innovation and adoption of job crafting to improve efficiency, sustainability, well-being, virtual teamwork, communication, and information sharing. The barriers to the adoption of job crafting were related to a multitude of organizational challenges, including a lack of human resource management practices, particularly training, and the characteristics of the technology used [16]. Additionally, a literature review from recent times concluded that innovation processes in healthcare systems are often obstructed by conflicts arising from conflicting elements, differing work cultures, and varying organizational and regulatory contexts. Since many of the tensions highlighted can be addressed, future policymaking and research should aim to develop strategies that manage these conflicts effectively, thereby improving the implementation of innovations [17].

Tan and Sim conducted interviews with 20 participants from seven enterprises in Singapore to explore their understanding and experiences of workplace innovation [5]. The findings revealed that despite the resource constraints typical of small and medium enterprises (SMEs), these organizations remained innovative to stay competitive and enhance their processes. The type of learning employees engage in significantly impacts their creative capabilities. Successful organizations foster both formal and informal learning environments to boost innovation. Notably, SMEs tend to favor work-integrated, incidental, and informal learning due to their limited resources. Additionally, it was observed that many enterprises regularly send their staff to training programs to keep them updated on the latest trends and practices that encourage innovation. However, Tan and Sim's study highlighted a deficiency in inter- and cross-organizational learning in public healthcare, which seems to impede innovation [5]. Similarly, Knutsson and Lurie also stated that weak leadership, coupled with limited knowledge of innovation, contributed to a general reluctance among the workforce to pursue improvements [13]. This reluctance stemmed from a shortage of time and expertise necessary to develop and implement new ideas. Consequently, employees struggled to generate and advance their own ideas effectively.

Leadership is among the structural barriers that limit employee innovation. The type of leadership differs concerning the type of care and facility provided [5]. Consultants did not like inter-organizational sessions to involve residence physicians within the same domain since it would cause problems in physician competition. The organization's size was also considered a crucial barrier because small organizations do not seem to guarantee workforce improvement in development and innovations, as seen in complex organizations. More prestigious departmental managers had long consultant experience. On the other hand, residence physicians were studying to become experts. Because the consultants had more power, the residence physicians were afraid to provide their ideas because they feared being viewed as annoying, promoting barriers to more ideas generation and innovation [5].

Communication is also a barrier to WDI. Evidence from studies indicates that the majority of respondents argued that their organizations lacked formal and informal forums to discuss innovations from workforce perspectives. Most healthcare systems deal with conservative high levels of hierarchy and structures that hamper communication. Tools for gathering ideas are lacking in an organization that lacks formal forums. Most healthcare with formal structures of sharing ideas were inconsistent and unsuccessful in communicating the goals and importance of innovation to the workforce [5]. However, according to Knutsson and Lurie, there is a noticeable semantic gap between the language used by the workforce and that of the innovation department. Both experts and employees noted that many workers do not view innovation as part of their responsibilities. Conversely, the innovation team was often perceived as ambiguous and complex. When addressing related issues with employees, the innovation team might use jargon that can create obstacles for those who are not well-versed in the topic [13].

Bardach et al. described that dedicated electronic health records IT and analytic support, as well as continual leadership engagement and communication, all contribute to the success of innovation. Flexible, yet guided, innovation support keeps teams accountable and motivated while meeting changing project demands and directions. Understanding the changing ecosystem and continuously analyzing and sharing outcomes allows teams to adapt as needed. Facilitation and support contribute to the value of varied, engaged teams; unique approaches and tactics generate innovative perspectives and encourage creative thinking [12].

Recommended Solutions

The scoping review by van den Hoed et al. outlined four major characteristics that contribute to innovation readiness in healthcare organizations: (i) Strategic course for innovation, (ii) Climate for Innovation, (iii) Innovation leadership, and (iv) Innovation commitment [18]. The factors influencing innovation readiness appear to correlate to those identified in the literature and are illustrated in Table 1.

**Table 1:** Factors contributing to innovation readiness Adapted From:  Table 2, van den Hoed et al., 2022 [18]; licensed under a Creative Commons Attribution 4.0 International License

Category	Key Components	Impact on Innovation Readiness
Strategic Course for Innovation	- Innovation strategy - Innovation program - Innovation process - Inter-organizational links	Provides a clear direction and structured approach to foster innovation readiness.
Leadership for Innovation	- Leadership style - Middle manager's role	Leadership shapes the culture and support needed to drive innovation.
Commitment to Innovation	- Innovative behavior - Innovative competencies	A strong commitment from staff promotes continuous innovation efforts.
Climate for Innovation	- Innovative organizational culture - Room for learning	A conducive climate encourages risk-taking and learning, boosting innovation.
Outcome	- Innovation readiness	The collective influence of these factors leads to a higher state of innovation readiness.

Resource-Based View (RBV)

Resource barriers, particularly for SMEs, are the main problem discussed in the literature that hinders workforce innovation. Gupta et al.'s study on how RBV promotes competitive advantage indicates that an organization's competitive advantage is generated by integrating a set of strategic internal resources [19]. RBV assumes that resources are varied and spread throughout the organization. As a result, it seeks to elucidate the relationship between sustained competitive advantage and resource composition. There is no doubt that healthcare organizations operate in complex and dynamic environments that impact organizational performance. As a result, RBV can help managers understand the degree of organizational efficiency within the internal environment to respond to external factors. While the organization can respond to the turbulent environment, it can create strategies that promote improved management. In the case of promoting employee innovation, managers are urged to take more control of resources and assume the role of gardeners. Gardening involves the managing of resources and human resources that need to be catered for and developed. Furthermore, the authors, Gupta et al., argue that the most significant resource for a firm operating in a dynamic environment is how well the knowledge and abilities are absorbed. Kash et al. discussed how one healthcare organization implemented a WDI strategy by creating a leadership development program [20]. This program was designed to cultivate managerial talent and enhance the decision-making capabilities of healthcare leaders. By focusing on leadership development, the organization was able to improve its responsiveness to external challenges such as regulatory changes and shifting patient demographics. This strategic investment in human capital not only empowered individuals within the organization but also fostered a culture of collaboration and innovation that permeated the entire system. The result was a more agile organization capable of adapting to new healthcare delivery models and improving patient outcomes, illustrating the RBV’s assertion that effective resource management, particularly the nurturing of leadership talent, can lead to a sustainable competitive advantage in a dynamic healthcare environment [20]. RBV also urges the promotion of strategic capabilities such as specific funding regimes that encourage the use of novel surgical approaches that are significant since they generate the potential to cater to more patients with minimal healthcare resources [19]. 

New Public Management

An NPM approach can be used to overcome the problem of top-down management. The literature indicates that most managers dominate decision-making and junior managers fear contributing their ideas due to their inferiority [21]. During the 1990s, NPM gained popularity across countries intending to hand over power from the politicians to managers by offering novel techniques for the management to deploy innovation for productivity increase reasons [13]. Failure to implement some NPM mechanisms such as incentives and competing markets created room for new management approaches to develop for example organizational entrepreneurship that has promoted bottom-up innovations. Bottom-up innovation demonstrates the inclusion of employees, managers, and systems that encourage innovation between knowledge sharing and agencies to promote good organization practices [13]. Knutsson and Lurie give evidence of the use of NPM across public sectors as an introduction to the market mechanism and customer responsiveness. This results in decentralization management, deregulation, and delegation. However, while NPM has brought about many benefits, its limitations in the healthcare sector must also be considered. For instance, deregulation and competition can sometimes lead to uneven service delivery, where the focus on cost-cutting measures may inadvertently compromise the quality of patient care. In some cases, NPM-driven reforms have prioritized financial efficiency over patient-centered outcomes, leading to concerns about the long-term sustainability of healthcare quality. Addressing these potential pitfalls ensures a more balanced view of NPM’s impact on healthcare innovation and management.

NPM characteristics are defined as autonomation, competition, deregulation, and disaggregation. Autonomation refers to granting more autonomy to lower-level managers and employees, empowering them to make decisions that drive localized innovation. In healthcare, this autonomy allows frontline staff, such as nurses and department heads, to develop and implement new processes or care models that meet the specific needs of their patients without waiting for top-level directives. Competition, another core element, is introduced through mechanisms such as privatization or performance-based incentives. In healthcare, this can encourage innovation as providers seek to differentiate themselves through improved patient care, cost efficiency, or service quality. Deregulation involves reducing the strict regulatory frameworks that often govern healthcare organizations, allowing them greater flexibility to experiment with new models of care or operational processes. Disaggregation involves breaking down large healthcare organizations into smaller, more manageable units. In practice, this can encourage innovation by creating more specialized, agile units that can focus on specific areas of care [13].

Employee Empowerment

Among the problems identified is the lack of managers promoting bottom-up ideas sharing and innovation. Tampi et al. argued that the only way an organization can survive in a competitive landscape is by having collaborative and proactive employees who share the organization's objectives. Healthcare is an organization with a high degree of human interaction. Therefore, empowerment can be critical to increasing employee job performance and satisfaction. Allowing the workforce to come up with innovative and new ideas can positively impact the organization. One of the most significant benefits of increased empowerment and freedom is that it promotes various employee concepts [22]. The diverse ideas presented by various participants within the organization can be understood through different perspectives. Beyond broadening the range of viewpoints, employee empowerment can also reduce managerial costs by transferring some responsibilities from managers to employees. Empowering employees is essential for enhancing their involvement in innovation and gaining valuable insights into related research areas [22]. Employee empowerment programs have been widely adopted to improve organizational performance. Some programs that can boost employee empowerment include providing rewards and offering employees access to job-related skills and knowledge [13]. For instance, in a government hospital in Abu Dhabi, the pharmacy staff were empowered to take the lead in improving patient wait times as part of an outpatient pharmacy Kaizen project [23]. This empowerment allowed staff to pilot ideas, make key decisions, and ultimately reduce wait times from 45-60 minutes to just four to six minutes, demonstrating how employee empowerment can drive innovation and lead to significant improvements in organizational performance.

This study provides a comprehensive analysis of the challenges hindering the effective implementation of employee-driven innovations, highlighting critical issues such as organizational resistance, insufficient support systems, and limited resources. By synthesizing existing research and presenting a thorough examination of these barriers, this review offers valuable insights that can guide healthcare leaders and policymakers in creating more supportive environments for innovation. Additionally, the review identifies actionable strategies to overcome these obstacles, thereby enhancing workforce engagement and contributing to the overall success and advancement of healthcare organizations. This focused approach not only deepens the understanding of WDI challenges but also fosters practical solutions to facilitate organizational improvement.

Limitations and Future Research Directions

In the context of this review, a key limitation is that, due to the emerging nature of this topic, there is a lack of extensive literature available for comprehensive comparison. Despite exhaustive database searches, the relatively nascent status of research in this field means that the review may not encompass all relevant studies or insights. Consequently, this limitation highlights the need for ongoing and future research to build a more robust understanding and address the gaps in current knowledge. There is a need for more comprehensive and longitudinal studies to track the evolution of WDI and the barriers encountered over time. Expanding research to include diverse geographic regions and healthcare systems could provide a more global perspective and reveal context-specific challenges. Additionally, investigating the impact of emerging technologies and new healthcare policies on WDI could offer valuable insights. Collaborative studies involving multidisciplinary teams might also enhance the analysis of complex barriers and propose more nuanced solutions. Finally, examining successful case studies and best practices from innovative healthcare settings can inform strategies to overcome identified obstacles and drive progress in this evolving field.

## Conclusions

This study highlights key barriers such as organizational resistance, insufficient support systems, and limited resources that hinder effective WDI. Addressing these barriers is essential for enhancing organizational performance and fostering a culture of innovation. Future research should focus on developing targeted interventions to overcome these barriers, examining the role of leadership and management practices, and exploring best practices across diverse healthcare settings. These insights will help create supportive environments for innovation and improve healthcare outcomes.
